# RETRACTION: GosB Inhibits Triacylglycerol Synthesis and Promotes Cell Survival in Mouse Mammary Epithelial Cells

**DOI:** 10.1155/bmri/9803274

**Published:** 2026-09-12

**Authors:** BioMed Research International

**RETRACTION**: G. Xu, S. Duan, J. Hou, Z. Wei, and G. Zhao, “GosB Inhibits Triacylglycerol Synthesis and Promotes Cell Survival in Mouse Mammary Epithelial Cells,” *BioMed Research International* 2017, no. 1 (2017): 1–12, 10.1155/2017/7394869

The above article, published online on 17 October 2017 in Wiley Online Library (wileyonlinelibrary.com), has been retracted by agreement between the journal Editor, Yoel A. Klug; and John Wiley & Sons Ltd.

The retraction has been agreed upon following an investigation into concerns raised by a third party and in PubPeer comments [1] regarding image inconsistencies. The flow cytometry plots presented in Figure 5(b) ‘Ad‐GosB’ appear to be reused from Figure 6 b ‘1’ in an earlier article by different authors [2]. Additionally, the cell image presented in Figure 2(a) ‘Control’ appears to be reused in Figure 4(a) ‘Control’. The flow cytometry plot presented in Figure 5(b) ‘Control’ appears to be reused in Figure 7(d) ‘Control’.

During the investigation, the authors failed to respond to any requests for explanations and did not provide original image data. The visual similarity and the absence of original image files undermines our confidence in the integrity of the article’s content and it is therefore retracted.

The authors were informed of the retraction, but did not respond.
